# The Involvement of the Endogenous Opioid System in the Gastrointestinal Aging in Mice and Humans

**DOI:** 10.3390/ijms23073565

**Published:** 2022-03-24

**Authors:** Agata Szymaszkiewicz, Marcin Talar, Jakub Włodarczyk, Mikołaj Świerczyński, Adrian Bartoszek, Julia Krajewska, Anna Mokrowiecka, Ewa Małecka-Wojciesko, Jakub Fichna, Marta Zielińska

**Affiliations:** 1 Department of Biochemistry, Faculty of Medicine, Medical University of Lodz, 92-215 Lodz, Poland; agata.szymaszkiewicz@stud.umed.lodz.pl (A.S.); marcin.talar@umed.lodz.pl (M.T.); jakub.wlodarczyk@stud.umed.lodz.pl (J.W.); mikolaj.swierczynski@stud.umed.lodz.pl (M.Ś.); adrianbartoszek96@gmail.com (A.B.); julia.krajewska@umed.lodz.pl (J.K.); jakub.fichna@umed.lodz.pl (J.F.); 2 Department of Digestive Tract Diseases, Medical University of Lodz, 90-153 Lodz, Poland; anna.mokrowiecka@umed.lodz.pl (A.M.); ewa.malecka-panas@umed.lodz.pl (E.M.-W.)

**Keywords:** aging, constipation, the elderly, endogenous opioid system, opioid receptors, opioids

## Abstract

Nearly 20% of elderly patients suffer from constipation, but the age-related changes in the gastrointestinal (GI) tract remain insufficiently elucidated. In this study, the alterations within the endogenous opioid system (EOS) as a potential cause of constipation in the elderly were evaluated. The GI functions were assessed in vitro and in vivo and compared between 6-, 12- and 18-month old mice. Moreover, the effect of opioid receptor (MOP, DOP, KOP) agonists on the mouse GI tract functions and the EOS components expression in mouse tissues and colonic biopsies from patients with functional constipation were determined. In the oldest mice, the GI peristalsis was significantly impaired as compared to the younger groups. The tissue response to MOP and DOP, but not KOP, agonists weakened with age in vitro; for DOP, it was confirmed in vivo. In the mouse upper GI tract, *Oprm1, Oprd1, Oprk1* expression decreased with age; in the colon, *Oprm1* expression increased. There were no differences in the expression of these genes in the colonic biopsies from patients >50 years old as compared to the younger group. In conclusion, the age-related impairment of the GI peristalsis may result from reduced MOP and DOP response to the activation with opioid agonists or the alterations in the EOS expression.

## 1. Introduction

Over the last decades, life expectancy has significantly increased; unfortunately, it has not been associated with a proportional improvement in patients’ quality of life but is linked with an increased risk of various diseases, dementia, or disabilities [1]. One of the most common and inconvenient problems in the elderly is GI peristalsis disturbance. Over 20% of advanced in years patients self-report constipation [2], and around 10% of people over 65 years old (y.o.) suffer from constipation and fulfill the Rome IV criteria for functional constipation (FC), constipation-predominant irritable bowel syndrome (IBS-C) or opioid-induced constipation (OIC) [3]. Numerous factors may contribute to constipation in seniors, i.e., imbalanced diet, dehydration, low level of physical activity, comorbidities, pharmacotherapy, or pelvic floor dysfunction. The alterations in the GI tract that occur with age, such as disturbances in the intestinal barrier permeability and smooth muscles contractility, as well as chronic age-related inflammation, disruptions in the brain-gut axis and in the enteric nervous system (ENS), may underlie the high prevalence of defecation impairment in the elderly [4,5].

The functions of the GI tract are influenced by the action of neuropeptides, including endogenous opioids: β-endorphins, Met- and Leu-enkephalins, and dynorphins [6]. These peptides, acting through the respective opioid receptors (ORs) μ-(MOP), δ-(DOP), and κ-(KOP), constitute the endogenous opioid system (EOS). Opioid receptors are widely expressed in the GI tract: on the neurons of submucosal and myenteric plexuses, smooth muscle cells, and on the terminals of the sympathetic and sensory peripheral neurons [7]. Opioids, especially these acting through MOP and DOP receptors, increase tonic and segmental contractions of the GI tract and thus impair relaxation of the lower esophageal sphincter, enhance tonus of pyloric and anal sphincters, decrease the propulsion of the smooth muscles in the intestines, prolong the gastric emptying and the GI transit time [8]. Furthermore, activation of DOP receptors stimulates absorption and impairs the secretion of water and electrolytes [7,9]. Due to the significant impact of opioidergic signaling on the GI tract functions, ligands of ORs are applied in the therapy of the GI diseases—e.g., eluxadoline (a mixed ligand of ORs, a drug approved for diarrhea-predominant irritable bowel syndrome (IBS-D) [10]), loperamide (a MOP agonist, applied in acute diarrhea; with limited application in IBS-D [11]), trimebutine (MOP, DOP, KOP agonist; applied in IBS [12]), methylnaltrexone (antagonist of peripheral MOP receptors that alleviates opioid-induced constipation [13]) or racecadotril (inhibitor of endogenous enkephalinases; applied in acute diarrhea [14]).

In this study, we aimed to expand our knowledge on the factors that contribute to constipation in the elderly concerning the EOS as an important component involved in the function of the GI tract. We hypothesized that the age-related alterations in the EOS might underlie the impairment of the GI peristalsis that occurs with age. 

## 2. Results

### 2.1. Gastrointestinal Peristalsis Was Impaired in Aged Mice

**Figure 1:**
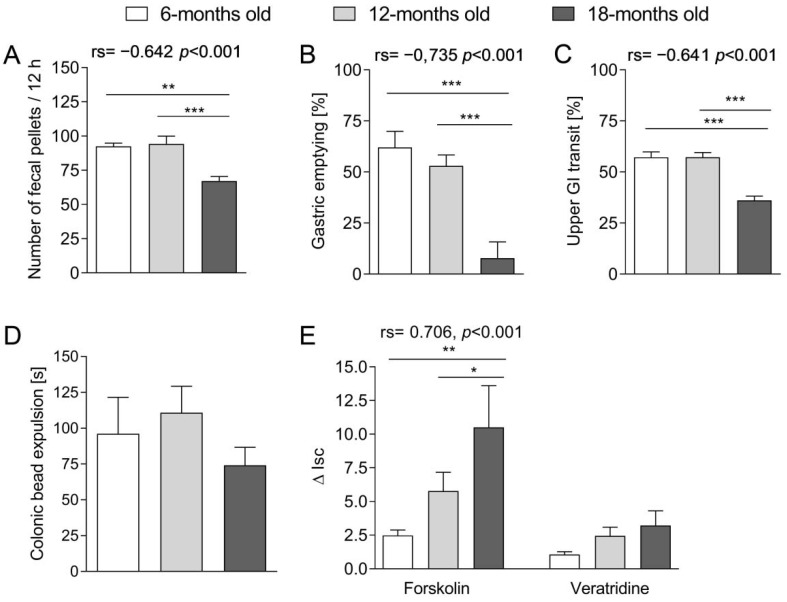
The evaluation of the age-related changes in the mouse GI tract. Panels from (**A**–**D**) present the alterations in the GI peristalsis: number of fecal pellets excreted over 12 h (**A**), gastric emptying (**B**), upper GI transit (**C**) and colonic transit (**D**) in 6-, 12- and 18-month old mice. (**E**) includes the comparison of the change in short circuit current (ΔI_sc_) between 6-, 12- and 18 month- old mice after stimulation with forskolin (10^−5^ M; left side of the figure) and veratridine (3 × 10^−5^ M, right side of the figure). One-way analysis of variance (ANOVA) followed by Bonferroni correction test was used to evaluate the difference between groups. Bars represent mean  ±  SEM. For significant association between age and given variables (**A**–**D**). Spearman’s rank correlation coefficient (denoted as rs) was presented. * *p* < 0.05, ** *p* < 0.01, *** *p* < 0.001.

We compared the body weight, intestinal length (small intestine and colon), and the number and weight of fecal pellets excreted overnight by 6-, 12- and 18-month-old (m.o.) mice (Table 1). The pellets were counted after 12 h, as mice are nocturnal and they remain the most active at night. We found that the intestine length (mainly colon) increased with age. Noteworthy, the number (Figure 1A) and weight of fecal pellets in the fecal pellet output test were significantly lower in the oldest group as compared to younger.

Next, we assessed the changes in the GI transit in different segments of the GI tract. We noted that gastric emptying decreased (Figure 1B), and the small intestinal transit was prolonged in 18-m.o. mice as compared to 6- and 12-m.o. animals (Figure 1C). We observed that both parameters correlated with mouse age, Spearman’s rank coefficient were −0.735 and −0.641, respectively. Interestingly, we found no statistically significant difference between groups in the colonic transit as assessed using the colonic bead expulsion test (Figure 1D).

### 2.2. cAMP-Dependent Secretion of Cl^−^ in the Colon Was Age-Dependent

In experiments with Ussing chambers, the addition of forskolin resulted in the activation of cAMP-dependent Cl^−^ secretion, which induced a rapid increase in short circuit current (I_sc_) that subsequently stabilized on a plateau. In our study, the sensitivity to the stimulation with forskolin differed significantly between the age groups, and ΔI_sc_ increased with age in response to forskolin (Figure 1E, left side). We also observed a strong positive correlation between ΔI_sc_ and the age of mice.

Veratridine stimulated ion secretion via activation of voltage-dependent Na^+^ channels. We found no difference in response to veratridine in tissues isolated from mice of different age groups (Figure 1E, right side). 

### 2.3. The Action of MOP and DOP Receptors Agonist Weakened with Age in the GI Tract

**Figure 2:**
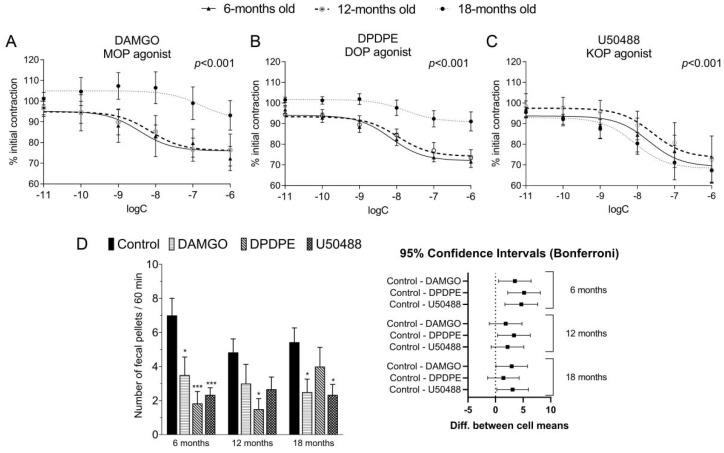
The action of ORs agonists in the GI tract of 6-, 12- and 18-month old mice. Top: dose-dependent effects of ORs agonists: DAMGO (0.1 mg kg^−1^) (**A**), DPDPE (0.3 mg kg^−1^), (**B**) and U50488 (1 mg kg^−1^) (**C**) on EFS-stimulated longitudinal smooth muscles contractility in the colon. Data are presented as mean ± SEM; n = 6–8 where n indicates the number of individual tissues from ≥3 different animals. The curves (**A**–**C**) were fitted to the three-parameter dose-response non-linear regression curve. *p* value was showed for comparing curves based on the extra-sum-of-squares F test. Bottom (**D**): the effect of DAMGO (0.1 mg kg^−1^), DPDPE (0.3 mg kg^−1^), and U50488 (1 mg kg^−1^) on 60 min fecal pellet output with calculated and graphed 95% Confidence Intervals for the difference of means compared to control (vehicle/placebo) group in right side. The results are expressed as mean ± SEM. One-way analysis of variance (ANOVA) followed by Bonferroni correction test was used to evaluate the difference between groups. * *p* < 0.05, *** *p* < 0.001 as compared to matched control.

We evaluated the effect of selective ORs agonists on the EFS-stimulated longitudinal smooth muscles contractility of the colon. Tissues were mounted in an organ bath under constant tension, and the stimulation with an electrical field resulted in rapid contraction (expressed as a change in tension of the tissue). Firstly, we observed that the addition of selective ORs agonists to the organ baths decreased the amplitude of initial longitudinal smooth muscle contractions in a concentration-dependent manner in all age groups (*p* < 0.001). Secondly, we found that the inhibitory action of DAMGO and DPDPE was significantly weaker in 18-m.o. mice as compared to younger groups (Figure 2A–C).

Subsequently, we evaluated the effect of selective ORs agonists on the GI peristalsis in vivo with the fecal pellet output test (Figure 2D). We noted that DAMGO (0.1 mg kg^−1^) and U50488 (0.3 mg kg^−1^) significantly reduced the number of fecal pellets excreted over 60 min in the groups of 6- and 18-m.o. mice as compared to controls. Interestingly, DPDPE (0.3 mg kg^−1^) impaired the fecal pellet output in 6- and 12-m.o. group as compared to control, and there was no significant effect in the oldest group.

### 2.4. Aging Led to Alterations in the Expression of the EOS Components at mRNA and Protein Levels

**Figure 3:**
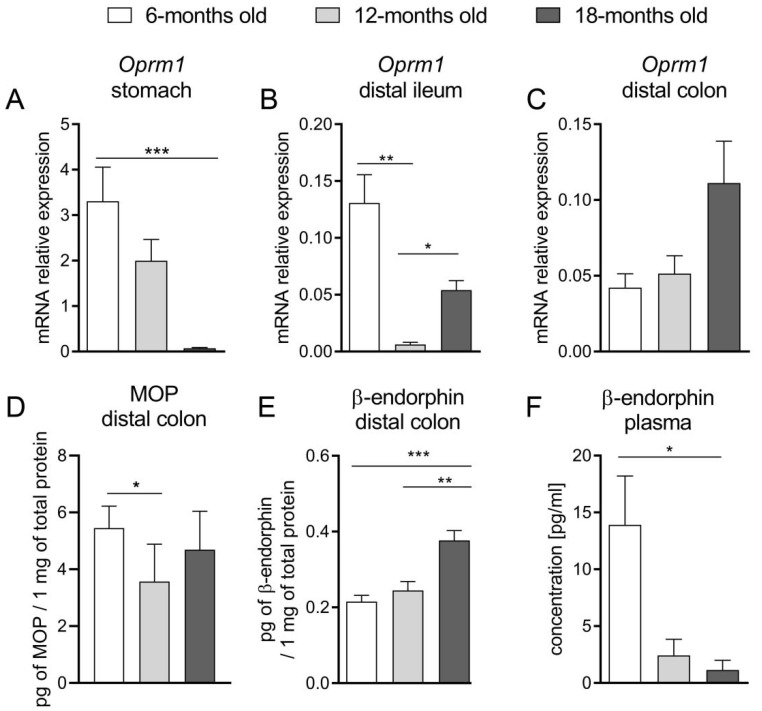
The mRNA expression of the *Oprm1* gene, encoding MOP receptor, in the stomach (**A**), distal ileum (**B**) and distal colon (**C**). Theprotein expression of MOP receptor (**D**) and β-endorphin (**E**) in the colon, the concentration of β-endorphin in the plasma (**F**) of 6-, 12- and 18-months old mice. Welch ANOVA test followed by Tamahane’s T2 multiple comparison test was used to evaluate the difference between groups (**A**–**C**). One-way ANOVA followed by Bonferroni correction test was utilized for multiple comparison in case of block (**C**,**D**). * *p* < 0.05, ** *p* < 0.01, *** *p* < 0.001.

With age, the mRNA expression of *Oprm1*, the gene encoding MOP receptor, was decreased in the stomach and distal ileum (Figure 3A,B). The *Oprm1* expression in the colon was not statistically significantly different as compared between groups (Figure 3C). At the protein level, the expression of the MOP receptor in the colon was slightly lowered in the 12-m.o. mice in comparison to 6-m.o. group but without any significant difference between the youngest and the oldest mice (Figure 3D). The concentration of β-endorphin in the colon was significantly increased in 12- and 18-m.o. mice as compared to 6-m.o. (Figure 3E), while in the plasma the opposite was noted: the concentration of this peptide was the highest in 6-m.o. old mice (Figure 3F). We also attempted to assess the mRNA expression of the gene encoding proopiomelanocortin, a β-endorphin precursor, but it was not sufficiently expressed to be detected in the whole mount tissue. 

**Figure 4:**
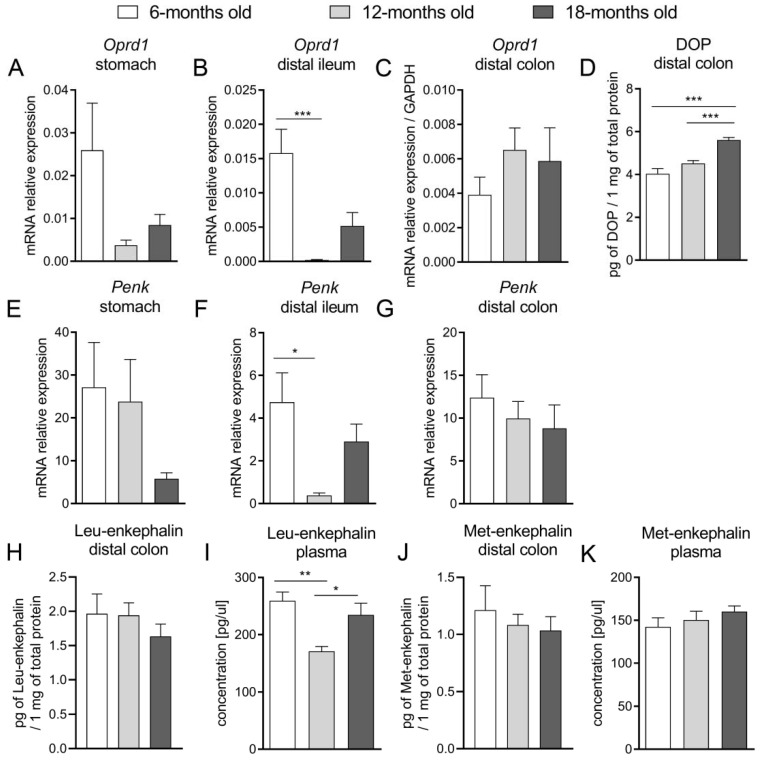
The mRNA expression of the *Oprd1* gene, encoding DOP receptor, in the stomach (**A**), distal ileum (**B**) and distal colon (**C**). The protein expression of DOP receptor (**D**) in the distal colon. The mRNA expression of *Penk* in the stomach (**D**), distal ileum (**E**) and distal colon (**F**). The protein expression of endogenous enkephalins: Leu-enkephalins (**H**,**I**) and Met-enkephalins (**J**,**K**) in the distal colon and in the plasma of 6-, 12- and 18-months old mice. Differences between groups were tested with one-way ANOVA followed by Bonferroni corrections for multiple comparisons apart from block (**A**) where Welch ANOVA test followed by Tamahane’s T2 was used, block (**C**,**G**) where Kruskal–Wallis test was applied with post hoc Dunn’s multiple comparisons test. * *p* < 0.05, ** *p* < 0.01, *** *p* < 0.001.

A similar tendency was observed for DOP receptors: in the stomach and distal ileum: the mRNA expression of *Oprd1* was significantly lower in 12- and 18-m.o. mice as compared to younger animals. Notably, in the colon, there was no statistically significant difference between groups (Figure 4A–C). A more apparent discrepancy was noted for the protein expression of DOP receptors in the distal part of the intestines: in the oldest group of mice, the protein expression of DOP receptors was higher than in 6- and 12-m.o. animals (Figure 4D). 

The differences in mRNA expression of *Penk*, which encodes enkephalin precursor, were subtle along the GI tract (Figure 4E–G). Similarly, there were no statistically significant differences in the expression of Leu- and Met-enkephalins in the colon (Figure 4H,J). The concentration of Leu-enkephalin in the plasma was lowered in 12-m.o. mice as compared to 6- and 18-m.o. (Figure 4I).

**Figure 5:**
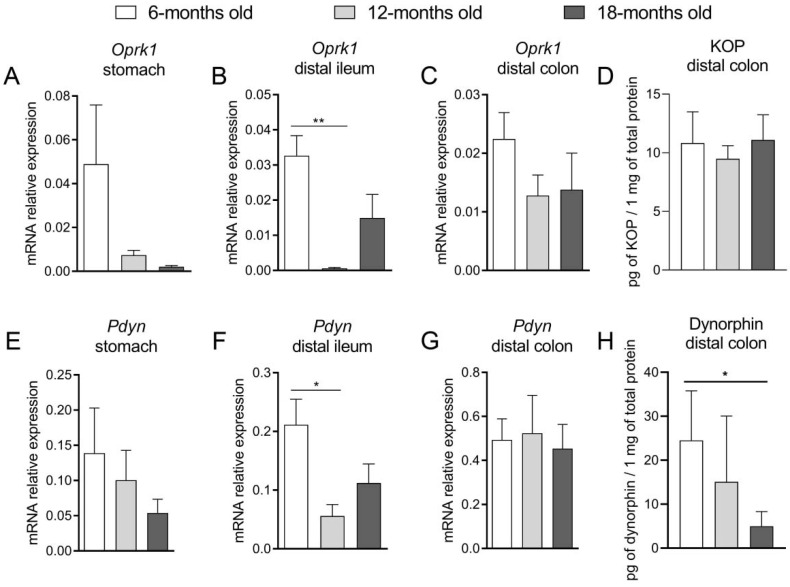
The mRNA expression of the gene *Oprk1*, encoding KOP receptor in the stomach (**A**), distal ileum (**B**) and distal colon (**C**). The relative protein expression of KOP receptor in the colon (**D**). The mRNA expression of *Pdyn* in the stomach (**E**), distal ileum (**F**) and distal colon (**G**) and the concentration of dynorphin in the colon (**H**) in 6-, 12- and 18-months old mice. One-way analysis of variance (ANOVA) followed by Bonferroni correction test was used to evaluate the differences between groups. Bars represent mean  ±  SEM. * *p* < 0.05, ** *p* < 0.01.

The expression of the *Oprk1* gene was significantly decreased in the distal ileum in 12-m.o. mice as compared to 6-m.o.; there were no statistically significant differences between groups in the stomach and in the colon (Figure 5A–C). There were no changes in the KOP receptor protein expression in the colon between groups (Figure 5D). Similar to *Oprk1*, the mRNA expression of *Pdyn*, a dynorphin precursor, was decreased in the distal ileum in the 12-m.o. mice as compared to the younger group; there were no significant differences between age groups in the stomach and the colon (Figure 5E–G). Noteworthy, the protein expression of dynorphin in the colon was significantly lower in the older mice as compared to the younger (Figure 5H). 

### 2.5. No Significant Difference in the mRNA Expression of the Genes Encoding ORs Were Found between the Patients with Functional Constipation >50 Years Old and Younger

**Figure 6:**
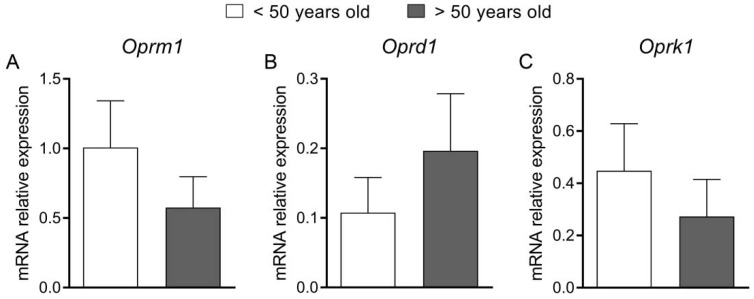
The mRNA expression of the *Opmr1* (**A**), *Oprd1* (**B**) and *Oprk1* (**C**) in the mucosal biopsies from the descending part of the colon from patients with constipation that were divided into two age groups: below 50 and over 50 years old. The difference between 2 groups was assessed with standard *t*-test, as the normality of data was not violated and variance homogeneity was proved.

In an attempt to translate the results from mouse models into patients, we evaluated the mRNA expression of the genes encoding ORs in the descending colon from patients with constipation (FC and IBS-C) and compared two age groups of patients (<50 and >50 y.o.). We did not notice any statistically significant differences between groups (Figure 6). 

## 3. Discussion

One of the issues that are attributed to the process of aging is a progressive impairment of the GI peristalsis. External factors, which contribute to constipation, are well known, while changes inside the intestines still constitute an unanswered question. In this study, we investigated the changes in the GI peristalsis and the transmural transport of ions through the intestinal wall associated with aging. We also aimed to investigate whether the alterations within the endogenous opioid system underlie the advanced age-related peristalsis impairment. 

In line with the literature on senescence [15,16], in our project, we chose three time-points of mouse life duration: 6-m.o. is considered as a young adult, 12-m.o. as an adult, and 18-m.o. as old. We confirmed the accuracy of the animal model used through the assessment of the intensity of the inflammatory state in the GI tract. As advanced age is associated with inflammation, which is defined as a chronic low-grade inflammation that develops in the elderly in the absence of infection [17,18], we measured the activity of myeloperoxidase (MPO) as a marker of inflammation. We found this parameter was the most pronounced in the oldest group of mice (data not shown). 

Then we attempted to evaluate the changes in the GI peristalsis that occurred with age. We showed that the number and weight of fecal pellets excreted decreased with age. A similar difference was observed in the transit of food content in the upper part of the GI tract: the stomach emptying and small intestine transit was prolonged in the oldest mice. Interestingly, we found no statistically significant difference between groups in the colonic transit. Therefore, we assumed that the differences in the number and weight of the excreted feces might be attributed to disturbances in the upper GI tract (stomach, small intestine) accompanied by the impaired absorption of water or imbalance of the process of secretion and absorption of fluids in the colon. Our observations were consistent with Patel et al. [19] on 3-, 6-, 18- and 24-m.o. C57BL/6J mice: they found that 24 h fecal pellet output decreased with age, and it resulted from a reduced number of pellets and the water content in the feces. However, they found that pellet propulsion in the colon decreased with age, which was not confirmed in our experiments and may be attributed to the differences in used animal models. In the study by Smits et al. [20], the gastric emptying and fecal pellet output, but not small intestinal transit, were significantly reduced in 24-m.o. rats in comparison to young (3-m.o.) and adult (12-m.o.) animals. 

The balance between the absorption and secretion of ions and water in the intestines constitutes an important component of the GI function. To expand our investigations on GI tract aging, we evaluated how transmural transport of ions changed with age in the colon. The first part embraced the assessment of the secretion in the tissue stimulated with forskolin (promotor of cAMP-dependent Cl^−^ secretion into the intestinal lumen). Forskolin added into the Ussing chamber stimulated the chloride ions transport through the intestinal wall, which was expressed as an increase in ΔI_sc_. We noted that the tissue sensitivity to the action of forskolin increased with age. According to our knowledge, this is the first study concerning the age-related alterations in this aspect in the mouse colon. We presume that lower sensitivity to the action of forskolin in younger mice may be the consequence of the high basal activity of chloride channels and the tissue resistance to further stimulation. In the older mice, there could be a gradual decline of basal activity of chloride channels, and therefore, the stimulation of forskolin entailed a more pronounced effect as compared to younger animals. The enhanced response to forskolin with age was previously noted in some organs, e.g., liver [21] or adrenal medulla [22]. The currently available data from the GI system involved the proximal part of the GI tract, and their results were contradictory to ours. Tuo et al. [23], who used a genetic model of premature senescence (based on the telomerase deficiency and severe telomere dysfunction), assessed the tissue response to forskolin in the stomach and the duodenum of prematurely aged mice and wild type animals. In telomerase-deficient mice (aged), the response to forskolin was significantly reduced as compared to wild types. 

In further experiments, we evaluated the impact of veratridine (a voltage-dependent Na^+^ channel activator) on the transmural ion transport in the colon. We observed that the sensitivity to the stimulation with veratridine did not differ between age groups and remained unchanged despite aging. As veratridine may increase the activity of the nitric oxide synthase (NOS) [24], Takahashi et al. [25] utilized the ability of NOS to produce nitric oxide (NO) and L-citrulline to indirectly evaluate the impact of aging on veratridine stimulation in the rat colon. It was observed that in the young animals (4–8-m.o.), stimulation with veratridine significantly enhanced NOS activity as compared to NOS basal activity, and there was no difference in aged rats (22–28-m.o.). Therefore, the tissue sensitivity to the stimulation with veratridine in the colon diminished with age. To summarize, our data showed that age-related changes in the GI tract encompass alterations in the transmural transport of ions in the intestines, namely: cAMP-dependent Cl^−^ secretion was affected by aging, while the action of voltage-dependent Na^+^ channel activator was not altered. 

Consequently, we studied the impact of ORs selective agonists on the EFS-stimulated intestinal contractility, and we confirmed that DAMGO, DPDPE, and U50488 significantly decreased the amplitude of longitudinal contractions of the colon in a concentration-dependent manner. Importantly, we found that tissue response to DAMGO and DPDPE, but not U50488, significantly decreased with age in vitro. In the past, the inhibitory effect of opioids on the EFS-stimulated smooth muscle contraction in the GI tract was evidenced in numerous studies on opioids [26,27,28,29], but none of them were focused on the aspect of aging. Interestingly, Broad et al. [4] analyzed functional changes that occur with age in the human colon. It was evidenced that in the colon, EFS-stimulated cholinergic signaling was attenuated in the elderly as compared to younger patients [4]. We assume that the attenuation of opioid activity with age observed in our study in vitro may be a combined effect of the age-related altered response to EFS and diminished response to opioids with age. 

We expanded our research by examining the effects mediated by opioids using fecal pellets output test in vivo. In this model, we observed attenuated activity of a DOP receptor agonist in the GI tract in 18-m.o. mice as compared to younger, similarly to our in vitro experiments. In contrast, there was no such effect in experiments with MOP receptors agonist DAMGO. Upon careful research of the literature, we found no reports on the influence of aging on the action of DOP receptors agonists in the GI tract. The findings only referred to the MOP receptor ligands. According to Vorsanger et al. [30], >65 y.o. patients treated with tramadol (MOP agonist and noradrenaline reuptake inhibitor) were more likely to suffer from constipation as compared to the younger group (28 vs. 17%). Due to the high clinical relevance of ORs agonists in pain management, our study was limited to the assessment of the action of agonists, not antagonists, in the GI tract. In clinics, the only antagonists that are applied in patients are peripherally acting antagonists of MOP receptors, such as methylnaltrexone [31] or naldemedine [32]. Noteworthy, it was reported that their activity in the human GI tract was independent of age [31,32].

To further explore this topic, we assessed the expression of the EOS components at mRNA and protein levels in the mouse GI tract. We noted that in the oldest mice, *Oprm1* expression in the stomach and ileum was significantly decreased. Furthermore, we did not find a statistically significant difference in mRNA expression of this gene in the colon, while the protein expression of MOP receptor in the 12-m.o. group was decreased as compared to younger mice. Noteworthy, there was an evident increment of the colonic β-endorphin protein expression in the oldest group of animals. The reduced expression of the MOP receptor may be a response to the enhanced production of β-endorphins in older mice. The age-related attenuation of the MOP receptor exogenous agonist (DAMGO) activity in the distal colon, which was discussed above, may be explained by the downregulation of MOP receptors in the colon. Furthermore, the higher level of β-endorphin, as an endogenous ligand of this receptor, noted in older groups, may lead to the persistent activation of these receptors and amplified MOP-related signaling. It could be expressed as delayed gastric emptying (as a consequence of increased tonus of pyloric sphincter) or impaired motility pattern of the intestine (reinforcement of tonic contractions and the reduction of propulsive waves). 

Concurrently, as the activation of DOP receptors increased the absorption of ions and water and impaired secretion, we suggested that alterations in the DOP receptor-related signaling would explain the changes in the functions of the GI tract observed in aged mice. We evaluated that mRNA expression of *Oprd1* decreased in the proximal part of the GI tract (stomach, ileum) with age, while there was no difference between groups in the colon. Noteworthy, protein expression of the DOP receptor was increased in older mice. At the same time, the mRNA expression of *Penk* was comparable in all age groups along the whole GI tract. Our results indicate that the molecular alterations regarding the expression of the DOP receptor and its endogenous ligands were subtle and difficult to correlate with pharmacological effects mediated by the exogenous agonist. However, we presume that the enhanced expression of DOP receptors could be a consequence of reduced DOP-related signaling observed in vitro and in vivo, but further studies focused on the activity of DOP receptors are required. 

In the past, Gerald et al. [33] measured the expression of the genes encoding EOS components in the mouse striatum and compared the results between two age groups (2–3 vs. 5–10-m.o.). Significant differences were found in the expression of *Oprm1* and *Oprd1*: in older male mice, the expression of the gene encoding MOP was lowered, while the expression of *Oprd1* increased with age. Furthermore, it was noted that the difference was not only in the mRNA expression of ORs but also the precursors of endogenous opioids: the expression of the genes encoding *Penk* was higher in female older mice, while expression of *Pdyn* increased with age in both sexes. 

Finally, we attempted to translate the results of the experiments on animal models into clinical conditions in patients with constipation (FC and IBS-C). Patients were divided into two age groups (<50 and >50 y.o.) and underwent a colonoscopy; descending colon biopsies were acquired, and the expression of mRNA of the genes encoding ORs was measured. We did not find any statistically significant differences between groups; however, the clinical part has several limitations. The most important limitation is the small number of patients in the groups (13 vs. 14 individuals). We found it difficult to recruit patients with constipation as the main ailment, as elderly patients usually have some important comorbidities that may contribute to constipation. Furthermore, we noted a significant discrepancy between the number of men and women in our study. We presume that it may be associated with a higher prevalence of FC in women: it was estimated at twice higher than in men [34]. According to Dothel et al. [35], who assessed the expression of MOP and β-endorphins in the mucosal biopsies of the descending colon of 31 patients with IBS (9 with IBS-C, 10 with diarrhea-predominant IBS, and 12 with mixed IBS), the mRNA and protein expression of MOP receptor and its endogenous agonist was increased in all IBS patients as compared to the asymptomatic control group. Among different IBS subtypes, the expression of β-endorphin was the lowest in IBS-C patients. Noteworthy, there were no differences in *Oprm1* expression between IBS patients. Our results cannot be compared with those obtained by Dothel et al. [35], as the age range is completely different: they included mainly young patients with IBS, aged between 30–40 y.o. and 35–45 y.o. individuals in the control group. 

To summarize, we performed a wide analysis of the alterations in the EOS that occur with age. We noted significant differences in the opioid receptors mediated effects in the GI tract in mice from different age groups. We presume that the changes within the EOS may be an important factor that contributes to the age-related peristalsis disturbance. Our results constitute a solid base for further studies on the involvement of the EOS in the age-related alterations in the GI tract with important clinical implications. 

## 4. Materials and Methods

### 4.1. Animals

In this study, male Balb/C mice (University of Lodz, Lodz, Poland) were used. The experiments were performed at 6-, 12- and 18-m.o. mice. Animals were housed in sawdust-lined plastic transparent cages, maintained under a 12-h light/dark cycle at 22–23 °C temperature. Mice had free access to tap water and laboratory chow. The whole project was performed in accordance to 3R role and respective national guidelines. All procedures were approved by the Local Ethical Committee for Animal Research (#47/ŁB73/2017).

### 4.2. Human Studies

The study was performed on patients of Caucasian origin (7 men and 20 women), who were admitted to Department of Digestive Tract Diseases at Medical University of Lodz, Lodz, Poland, from March 2018 to July 2020. Protocol was approved by The Bioethical Commission of Medical University of Lodz (RNN/288/17/KE). Patients were allocated to two groups, younger than 50 y.o. (n = 13; 12 women, 1 man) and older than 50 y.o. (n = 14, 8 women, 6 men). Patients fulfilled the Rome IV criteria for either IBS-C or functional constipation (FC). After admission to The Department of Digestive Tract Diseases, they underwent colonoscopy for typical clinical indications or as colorectal cancer screening program. Colonoscopies results were normal in all of them. During colonoscopy, one or two mucosal samples from the left-side colon were taken, frozen, and stored until further use. 

### 4.3. Reagents and Drugs

All drugs and reagents, unless otherwise stated, were purchased from Sigma-Aldrich (Poznan, Poland). Opioid agonists: DAMGO, DPDPE, U50488, were purchased from Tocris Bioscience (Warsaw, Poland).

### 4.4. Aging of the Gastrointestinal Tract

All of the experiments described below were conducted on mice of different age groups (6-, 12- and 18-m.o.).

#### 4.4.1. In Vivo-Fecal Pellet Output Test

Non-fasted mice were separated and placed into individual cages for 12 h. After this period, the fecal pellets excreted overnight were counted and weighed. On the next day, respective agonist of ORs (DAMGO, 0.1 mg kg^−1^; DPDPE, 0.3 mg kg^−1^; U50488, 1 mg kg^−1^) or vehicle (5% DMSO in 0.9% NaCl) were administered intraperitoneally (i.p.). The doses of ORs agonists were chosen according to the results of preliminary experiments. Immediately after injection, animals were placed in individual cages, and fecal pellets, excreted over a 60 min period, were counted. The results were compared between groups.

#### 4.4.2. In Vivo-Upper Gastrointestinal Tract

The transit of the upper part of the GI tract was assessed in mice fasted overnight. On the experimental day, animals received 0.2 mL of a red marker solution (50 mg phenol red in 100 mL 1.5% methylcellulose) via gastric gavage. Mice were sacrificed after 20 min from the marker administration; the stomach and small intestine were gently isolated. The distance from the pyloric sphincter to the front of the marker was measured and expressed as percent of the total length of the small intestine. 

#### 4.4.3. In Vivo-Gastric Emptying

The stomach was isolated as described above. After the isolation, it was opened, and the content was transferred to a plastic tube containing 4 mL of distilled water. After 20 min of sedimentation, 1 mL of supernatant was transferred to another tube containing 1 mL of 1 M NaOH to obtain the maximal intensity of the color. The solutions were colorimetrically assayed with iMARK Microplate Reader, Biorad, UK at 560 nm, and the gastric emptying was calculated according to the following formula:

$$\mathrm{GE}=100\%\;\times \left(1-\frac{\mathrm{amount}\;\mathrm{of}\;\mathrm{phenol}\;\mathrm{red}\;\mathrm{after}\;20\;\min}{\mathrm{amount}\;\mathrm{of}\;\mathrm{phenol}\;\mathrm{red}\;\mathrm{after}\;0\;\min}\right)$$

#### 4.4.4. In Vivo-Colon Beads Expulsion Test

The distal colonic expulsion test was performed in mice that were fasted for 12 h. On the experimental day, each mouse was slightly anesthetized by inhalation of 2% isoflurane (Aerrane, Baxter, Deerfield, MA, USA), and then, a pre-warmed (37 °C) glass bead was inserted into the distal colon. Right after, animals were placed in individual cages on a white sheet, and time to bead expulsion was measured. 

#### 4.4.5. Ex Vivo-Epithelial Ion Transport

The epithelial ion transport was evaluated according to the method described previously [36]. Briefly, 1 cm sections of the distal colon were isolated and placed in Ussing chambers (Physiologic Instruments, Inc., San Diego, CA, USA) filled with 6 mL of Krebs buffer (NaCl, 115 mM; KH_2_ PO_4_, 2 mM; MgCl_2_, 2.4 mM; NaHCO_3_, 25 mM; KCl, 8 mM; CaCl_2_, 1.3 mM). The solution was saturated with 95% O_2_ and 5% CO_2_ at a constant temperature (37 °C) and supplemented with either glucose (10 mM) or mannitol (10 mM) by the basolateral and mucosal side, respectively. The exposed area of the tissue was 0.3 cm^2^. Tissues were voltage-clamped to zero, using the WPI EVC-4000 voltage-clamp apparatus (World Precision Instruments, Sarasota, FL, USA) with Ag/AgCl electrode and 3 M KCl agar bridge. Once a stable baseline in short circuit current (Isc, mA cm^−2^) was reached (15–30 min), tissues were challenged with either forskolin (FSK) (10^−5^ M, cAMP-dependent secretagogue) or veratridine (VER) (3 × 10^−5^ M, voltage-dependent Na^+^ channel activator). For each challenge, the peak change in Isc (∆Isc) was established.

#### 4.4.6. In Vitro-Isolated Smooth Muscles Contractility

Mice were sacrificed by cervical dislocation. Subsequently, the colon was gently removed and full-thickness fragments (0.5 cm) of the distal colon were put in Krebs solution (NaCl 115.0 mM, KCl 8.0 mM, KH_2_PO_4_ 2.0 mM, NaHCO_3_ 25.0 mM, MgCl_2_ 2.4 mM, CaCl_2_ 1.3 mM, glucose 10.0 mM). One end of each colonic fragment was attached to the bottom of the individual organ bath, another to an FT03 force-displacement transducer (Grass Technologies, West Warwick, RI, USA) using a silk thread. The colonic segments were placed between two electrodes in an organ bath containing Krebs solution (25 mL), constantly oxygenated with 95% O_2_ and 5% CO_2_ at 37 °C. The changes in tension were amplified by a P11T amplifier (Grass Technologies, West Warwick, RI, USA) and recorded. Electrical field stimulation (EFS) was applied by an S88X stimulator (Grass Technologies, West Warwick, RI, USA; EFS, 8 Hz, 60 V, pulse duration 0.5 ms, train duration 10 s) and delivered through electrodes placed around the tissue to induce colonic contractions. The preparations were exposed to the action of increasing concentrations of selective ORs ligands: DAMGO, DPDPE, U50488 (10^−11^–10^−6^ M, 8 min for each concentration). The effect of tested compounds was recorded on a computer using the PolyView software (Polybytes Inc., 108 Cedar Rapids, IA, USA). The mean amplitude of four twitch contractions was measured and treated as an internal control. The changes in smooth muscle contractions were reported as the percentage of the internal control. These assays were performed as paired with respective controls, using four organ baths in parallel. The results for each agonist were compared between mice from different age groups (6-, 12- and 18-m.o.).

### 4.5. Mouse Blood Collection and Plasma Preparation

The cardiac puncture was performed according to procedure described by Parasuraman et al. [37]. Mice were anesthetized with 100 μL of a ketamine/xylazine solution (1 mL of ketamine, 0.5 mL of xylazine diluted in 8.5 mL of 0.9% NaCl), and held in a vertical position. Blood samples were collected directly from the heart chambers using a disposable needle (27G) with syringe (max volume 1 mL). Blood was taken slowly in order to avoid collapsing of the heart. Right after the blood collection, mice were sacrificed by cervical dislocation. Blood was stored in EDTA-container for 4 h at 4 °C, and then it was centrifuged at 3000 rpm for 3 min at 4 °C and then again for 1 min. Aliquots were stored at −80 °C for further use.

#### 4.5.1. Mouse Tissue Collection

The segments from the stomach, distal ileum, and distal colon of each animal were resected. Food/fecal contents and connective tissue residues were gently removed, and the tissue samples were rinsed with PBS. The colon samples were transferred to new tubes and stored at −80 °C for further analysis. 

#### 4.5.2. Sample Homogenization for Protein Analysis

Briefly, tissues were minced and homogenized with a bead homogenizer (Precellys Evolution, Bertin, France) in cell lysis buffer (1M Tris-HCl, 4M NaCl, 10% Triton X-100, H_2_O, and complete protease inhibitor cocktail). Then, the content was centrifuged at 14,000 rpm for 20 min at 4 °C. Each supernatant was diluted to the total protein concentration of 2 ng ml^−1^ and kept at −80 °C for further analysis. 

#### 4.5.3. ELISA Assays

The protein expression of β-endorphin (Cusabio, Wuhan, Hubei Province, China; CSB-E06827m), Leu-enkephalin (Mybiosource, San Diego, CA, USA, MBS755907), Met-enkephalin (Mybiosource, San Diego, CA, USA; MBS756178), dynorphin (Mybiosource, San Diego, CA, USA, MBS021366), MOP receptor (LSBio, Seattle, WA, USA; LS-F15095), DOP receptor (Mybiosource, San Diego, CA, USA; MBS7234849), KOP receptor (Abbexa, Cambridge, United Kingdom; abx156998) were assessed in the plasma and colon according to the protocol provided by producer. The specificity of the antibodies used in ready-to-use ELISA kits was declared by the manufacturers, and the authors did not further assess it. Therefore, the possible cross-reactivity between peptides cannot be excluded. 

#### 4.5.4. RNA Isolation

Samples were isolated using a Total RNA Mini kit (A&A Biotechnology, Gdynia, Poland) according to the manufacturer’s protocol. The purity and quantity of the isolated RNA were measured using a Colibri Microvolume Spectrometer (Titertek Berthold, Colibri, Germany). 

#### 4.5.5. Reverse Transcription

Total RNA (2 μg) was transcribed with cDNA with High-Capacity cDNA Reverse Transcriptase Kit (Life Technologies, Carlsbad, CA, USA).

#### 4.5.6. Quantitative Real-Time RT-PCR

For the quantification of mRNA expression in mouse tissue, we used the real-time fluorescence detection PCR method with FAM dye-labeled TaqMan probes: *Oprm1* (Mm01188089_m1), *Oprd1* (Mm01180757_m1), *Oprk1* (Mm01230885_m1), *Pomc* (Mm00435874_m1), *Pdyn* (Mm00457373_m1), *Penk* (Mm01212875_m1). Values obtained for studied genes were normalized to the expression of the glyceraldehyde 3-phosphate dehydrogenase (*Gapdh*, Mm99999915_g1, Thermofisher, Waltham, MA, USA) as an endogenous control. 

The mRNA analysis of human samples was performed using the following FAM dye-labeled TaqMan probes (Thermofisher, Waltham, MA, USA): *Oprm1* (Hs01053957_m1), *Oprd1* (Hs00538331_m1), *Oprk1* (Hs00175127_m1), and *HPRT1* (Hs1003267_m1) as an endogenous control. 

The cDNA was amplified in a LightCycler (Roche, Switzerland). The cycle parameters were as follows: initial denaturation at 95 °C for 10 min, followed by 55 cycles of sequential incubations at 95 °C for 15 s and at 60 °C for 1 min. The initial amount of the template was evaluated as a Ct parameter. The Ct (cycle threshold) values for all tested genes were normalized to Ct values obtained for housekeeping gene. Relative amount of mRNA copies was calculated using the following equation: 2^[−(Ct tested gene^^–^^Ct Reference gene)]^ × 1000.

### 4.6. Statistics

Statistical analyses were performed using the following software package: Prism v 9.0 (GraphPad Software Inc., La Jolla, CA, USA). The data are expressed as means ± SEM (Standard Error of Mean) as a measure of the dispersion of outcomes.

Assumption of Gaussian data distribution was evaluated with Shapiro–Wilk test. Variance homogeneity was tested with Levene’s test. In case of normal data, we performed PRISM ROUT test (Q = 1%, determines how aggressively the method will remove outliers) to identify extremely deviant scores as their occurrence and extremity substantially reduce probability of Type 1 error and decline statistical power (increase the probability of Type 2 error) in parametrical test.

The significance of differences was tested with Student’s t test to compare two independent groups.

For multiple comparisons, a one-way analysis of variance with Bonferroni’s correction was employed to determine the significance of differences between groups with distributions not departing from normality. In the case of unequal variances, Welch’s correction was applied, and conservative T2 Tamahane’s post hoc test was chosen accordingly. When the normality assumption was violated, the significance of differences was tested with the Kruskal–Wallis test followed by post hoc Dunn’s test.

In order to assess the degree to which two continues variables were related, Spearman’s rank correlation coefficient (abbreviated as rs) was used.

Dose-response curves for ORs agonists were fitted by non-linear regression (the log (inhibitor) vs. response equation, standard slope model). The extra-s um-of-squares *F* test was used to compare curves for the groups of 6-, 12- and 18-months old mice.

The null hypothesis in all calculations was rejected if *p* < 0.05.

## Figures and Tables

**Table 1:** The comparison of body weight, length of the intestines, the number and weight of the fecal pellets excreted overnight between 6-, 12- and 18-m.o. mice. Data are mean ± SEM. n = 7–9. rs * denotes Spearman’s correlation coefficient.

Age [Months]	Body Weight [g]	Total Length of the Intestine [cm]	Small Intestine Length [cm]	Colon Length [cm]	Number of Pellets/12 h [g]	Fecal Pellets Weight [g]
				rs * = 0.700, *p* < 0.001	rs * = −0.642, *p* < 0.001	rs * = −0.501, *p* < 0.001
6	29.2 ± 1.3	58.7 ± 2.6	49.2 ± 2.2	9.5 ± 1.0	91.9 ± 2.8	1.398 ± 0.093
12	32.1 ± 3.0	56.6 ± 3.3	46.6 ± 3.1	10.0 ± 0.5	91.0 ± 5.4	1.216 ± 0.126
18	32.6 ± 1.9	61.3 ± 2.6	50.2 ± 2.7	11.1 ± 0.5	65.3 ± 3.3	1.026 ± 0.125

## Data Availability

The data presented in this study are available on request from the corresponding author.
